# Delayed mammography screening and advanced breast cancer: variation across age and income groups

**DOI:** 10.1186/s12889-026-27731-4

**Published:** 2026-05-12

**Authors:** Yu-Hsun Wang, Nai-Chen Shih, Ying-Cheng Chen, Chao-Bin Yeh

**Affiliations:** 1 Institute of Medicine, Chung Shan Medical University, Taichung, Taiwan; 2 Department of Medical Research, Chung Shan Medical University Hospital, Taichung, Taiwan; 3 Department of Family Medicine, Taichung Veterans General Hospital, Taichung, Taiwan; 4 Department of Surgery, Changhua Christian Hospital, No. 135 Nanhsiao Street, Changhua, 500 Taiwan; 5 Department of Post-Baccalaureate Medicine, College of Medicine, National Chung Hsing University, Taichung, Taiwan; 6 Department of Emergency Medicine, School of Medicine, Chung Shan Medical University, No. 110, Sec. 1, Jianguo N. Rd, Taichung, 402 Taiwan; 7 Department of Emergency Medicine, Chung Shan Medical University Hospital, Taichung, Taiwan

**Keywords:** Mammography, Screening interval, Breast cancer, Case-case study

## Abstract

**Background:**

Regular screening mammography can detect breast cancer at an earlier stage, leading to better outcomes, including significantly improved survival rates and quality of life. However, the association between delayed mammography and the likelihood of advanced-stage breast cancer remains unclear.

**Methods:**

This population-based case-case study used Taiwan’s National Health Insurance Research Database linked to the Cancer Registry to examine the association between delayed mammography and advanced-stage breast cancer. Subjects with stages 0–II breast cancer were classified as early-stage, while those with stages III–IV were classified as advanced-stage breast cancer. The main exposure was time since last screening mammogram prior to cancer diagnosis. Multivariable logistic regression was used to estimate odds ratios (ORs) and 95% confidence intervals (CIs) for advanced cancer, adjusting for demographic and clinical factors.

**Results:**

11,672 were diagnosed with early-stage breast cancer, and 2,220 with advanced-stage breast cancer. Compared with patients who had undergone mammography screening, those who had never undergone screening had higher odds of being diagnosed with advanced-stage breast cancer (aOR = 1.94, 95% CI = 1.73–2.16). Compared with women with more recent mammography, those with screening intervals of more than two years (aOR = 1.42, 95% CI = 1.18–1.70) and more than four years (aOR = 1.38, 95% CI = 1.13–1.68) had higher odds of advanced-stage breast cancer. Age-specific analyses showed that delaying mammography over four years was associated with higher odds of advanced-stage breast cancer in women aged 40–69, those 70 and older, and in low-income groups.

**Conclusions:**

Delayed mammography screening is associated with higher odds of advanced-stage breast cancer at diagnosis, particularly among women aged 40 and older and those in low-income groups.

**Supplementary Information:**

The online version contains supplementary material available at 10.1186/s12889-026-27731-4.

## Introduction

Breast cancer is the most commonly diagnosed cancer and a leading cause of cancer death among women worldwide [1]. Early detection through screening mammography has been shown to reduce breast cancer mortality by allowing treatment at an earlier stage of the disease [2, 3]. Current guidelines recommend screening mammography for average-risk women starting at age 40 years [4–6]. In Taiwan, the Health Promotion Administration provides publicly funded biennial screening mammography for women aged 40 to 74 years. However, the breast cancer screening rate among women in Taiwan remains relatively low at approximately 40% [7]. This suboptimal participation rate highlights the importance of identifying factors associated with delayed or missed screening. In most healthcare systems, primary care physicians (often the most frequent point of contact for women) serve as coordinators of preventive services, where mammography arrangement, reminders, and follow-up predominantly occur [8]. Physician recommendations remain the strongest predictor of adherence. In addition, shared decision-making supported by patient decision aids—such as printed pamphlets or web-based interactive tools designed to help women understand the benefits, risks, and personal implications of breast cancer screening—is essential in primary care to reduce decisional conflict and improve screening intentions [9, 10].

Delayed or missed screening likely contributes to a substantial proportion of breast cancers being diagnosed at an advanced stage. A population-based study in the Netherlands demonstrated that women who did not attend mammography screenings had a significantly higher likelihood of being diagnosed with stage IV breast cancer compared to those who participated in screenings [11]. Additionally, research indicates that delays from initial presentation to diagnosis are associated with higher cancer stages and poorer survival rates [12].

While the importance of routine screening mammography is well established, previous studies have primarily focused on the delays in treatment for women diagnosed with breast cancer [13–15]. From a primary care perspective, delayed mammography extends beyond individual patient factors; it may reflect gaps in preventive care quality, including inadequate follow-up, communication deficits, and failure to identify or address socioeconomic barriers [16, 17]. To our knowledge, this is the first population-based study in Taiwan to examine the association between specific mammography screening intervals and advanced-stage breast cancer at diagnosis, with particular focus on age and income disparities. It is noteworthy that few studies have quantified the relationship between delayed mammography and stage at diagnosis, which may serve as an important population-level indicator of primary care effectiveness in cancer prevention. This study aims to investigate the association between delayed mammography and advanced-stage breast cancer, utilizing a retrospective population-based case-case design. Examining this relationship aims to emphasize the necessity of timely screening and its crucial role in improving breast cancer outcomes.

## Methods

### Data source

The Taiwan National Health Insurance Research Database (NHIRD) contains claims data on over 99% of the population in Taiwan. The NHIRD provided longitudinal records of outpatient visits, hospitalization, diagnosis of disease, prescription of medicine, surgical codes, and screening tests, as well as individual insured regions and insured salaries. The database was managed by the Health and Welfare Data Science Center (HWDC). The Longitudinal Health Insurance Database (LHID), which comprises two million beneficiaries randomly sampled from the registry of the entire Taiwanese population, encompasses data from 2000 to 2021. We linked the LHID to the Taiwan Cancer Registry to identify breast cancer stage diagnosed between 2007 and 2021. The cancer registry provided clinical data including initial diagnosis date, age at diagnosis, primary site, tumor size, chemotherapy, radiotherapy, and surgical treatments, histological type, clinical staging, and pathological staging according to the American Joint Committee on Cancer (AJCC). The study received approval by the ethical review board of the Chung Shan Medical University Hospital (CS1-22090).

### Study design and participants

This study was conducted by using the retrospective case-case study design. The study population was sourced from the Taiwan Cancer Registry, involving the selection of female individuals aged 18 and above diagnosed with breast cancer (International Classification of Diseases for Oncology, 3rd Edition, ICD-O-3 codes = C50) between 2007 and 2019. To ensure the inclusion of newly diagnosed breast cancer cases, individuals previously diagnosed with other types of cancer before their initial breast cancer diagnosis date were subsequently excluded. The early-stage group was defined as patients with breast cancer staged 0–II. The advanced-stage group consisted of patients with breast cancer staged III–IV. Cancer staging was initially classified based on clinical tumor staging, with pathological staging used if clinical staging information was unavailable. The index date for both groups was defined as the date of the initial breast cancer diagnosis.

### Mammography measurement

The American Cancer Society recommends that women aged 40 to 44 have the option for annual mammograms, while those aged 45 to 54 should get them yearly. Women 55 and older can switch to biennial screenings. All women should understand the mammogram process, including its benefits and limitations [5]. In Taiwan, the Health Promotion Administration provides publicly funded subsidies for biennial mammography screening. Prior to policy expansion, the program targeted women aged 45 to 69 years. Additionally, women aged 40 to 44 years with a family history of breast cancer—specifically in first- or second-degree relatives, including mothers, daughters, sisters, and grandmothers on either the paternal or maternal side—were also eligible for biennial screening. As of 2025, the eligibility criteria have been expanded to include women aged 40 to 74 years, reflecting updated national screening recommendations aimed at improving early detection of breast cancer [18].

Mammography exposure was identified from the LHID using the claim code 33005B. The screening period considered was from 2000 until the index date. To differentiate the delay interval in mammography exposure, the study categorized the time from the index date to the last mammography (or no prior mammography) into four groups: no mammography, less than or equal to two years, three to four years, and more than four years.

### Definition of covariates

The baseline characteristics were age, region, monthly income, and comorbidities. The classification of region and monthly income is based on the individual’s insured region and the insured salary at the index date. Residential areas are categorized according to Taiwan’s geographical distribution into northern, central, southern, eastern regions, and outlying islands (Supplementary Table 1). Monthly income levels are categorized into low (less than NT$25,000), medium (NT$25,000 to NT$39,999), and high (NT$40,000 or more). The comorbidities included hypertension, hyperlipidemia, chronic liver disease, chronic kidney disease, diabetes mellitus, chronic obstructive pulmonary disease, rheumatoid arthritis, ankylosing spondylitis, ischemic heart disease, stroke, hepatitis B, hepatitis C, obesity, benign mammary dysplasia, other disorders of breast, benign neoplasm of breast (Supplementary Table 2). Those comorbidities were defined between 2000 and index date and at least three outpatient visits or once hospitalization.

### Statistical analysis

The comparison of early-stage group and advanced-stage group were performed by Chi-square test and Student’s T-test were used for categorical and continuous variables, respectively. Univariate and multivariate logistic regression analyses were used to estimate the association between the mammography delay interval and the odds of advanced-stage breast cancer. The multivariate logistic regression analyses were adjusted for the following covariates: age, hypertension, hyperlipidemia, chronic liver disease, chronic kidney disease, diabetes mellitus, chronic obstructive pulmonary disease, ischemic heart disease, stroke, obesity, benign mammary dysplasia, other disorders of the breast, and benign neoplasm of the breast. To assess the robustness of our findings, we conducted sensitivity analyses using a matched design in which patients with advanced-stage breast cancer were matched to those with early-stage disease on age, region, income level, and year of diagnosis. This approach was intended to reduce potential confounding by demographic and temporal factors.

In addition, stratified analyses were conducted according to age and monthly income levels. Age was categorized into three groups: <40 years, 40–69 years, and ≥ 70 years. Monthly income was grouped into low (< NT$25,000), medium (NT$25,000–39,999), and high (≥ NT$40,000). These income categories were defined to reflect meaningful socioeconomic strata in Taiwan, approximating low-, middle-, and higher-income groups based on the distribution of insured payroll and national wage levels. These stratified analyses further assessed the association between the mammography delay interval and the risk of advanced-stage breast cancer within each subgroup. The SAS version 9.4 (SAS Institute Inc, Cary, NC, USA) was used for the statistical analysis. The p value less than 0.05 was defined as statistical significance.

## Results

### Baseline characteristics of the study group

**Fig. 1:**
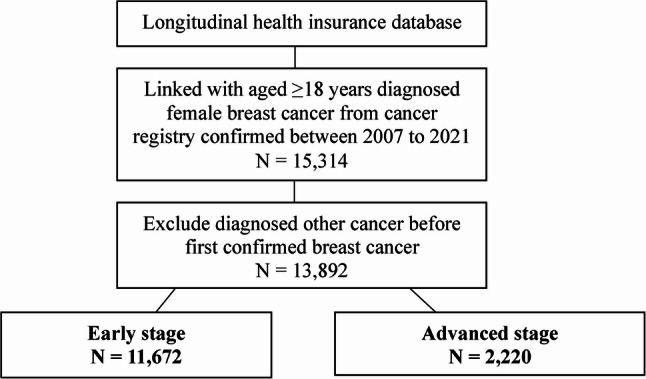
Flow-chart of subject selection

From 2007 to 2021, this study included 15,314 female breast cancer patients aged 18 years and older from cancer registry. After excluding those with previous cancers, a total of 13,892 patients were included in the final analysis. Further cancer staging categorization resulted in 11,672 patients classified as early stage and 2,220 patients as advanced stage (Fig. 1). The demographic characteristics are summarized in Table 1. Most early-stage patients were aged 40 to 69 years (79.80%), while advanced-stage patients were also predominantly in this age group (76.85%). The mean age for early-stage patients was 54.24 years with a standard deviation of 11.85, while for advanced-stage patients, the mean age was 56.58 years with a standard deviation of 12.77. The geographical distribution is mainly concentrated in the northern region, followed by the southern region, but there is no statistically significance in the geographical distribution of early-stage and advanced-stage breast cancer. In the distribution of monthly income, advanced-stage breast cancer tends to have a higher proportion among those with a low monthly income.

**Table 1:** Demographic characteristics of breast cancer

	Early-stage	Advanced-stage	*p* value
	*N* = 11,672	*N* = 2220	
Age			< 0.001
18–39	1203 (10.31)	171 (7.70)	
40–69	9314 (79.80)	1706 (76.85)	
≥ 70	1155 (9.90)	343 (15.45)	
Mean ± SD	54.24 ± 11.85	56.58 ± 12.77	< 0.001
Region			< 0.001
Northern	6329 (54.22)	1059 (47.70)	
Center	2171 (18.60)	424 (19.10)	
Southern	2702 (23.15)	625 (28.15)	
Eastern	417 (3.57)	105 (4.73)	
Outlying islands	53 (0.45)	7 (0.32)	
Monthly income (NT$)			< 0.001
Low	7256 (62.17)	1546 (69.64)	
Medium	1993 (17.08)	342 (15.41)	
High	2423 (20.76)	332 (14.95)	
Comorbidities			
Hypertension	3451 (29.57)	737 (33.20)	0.001
Hyperlipidemia	3283 (28.13)	519 (23.38)	< 0.001
Chronic liver disease	1750 (14.99)	258 (11.62)	< 0.001
Chronic kidney disease	250 (2.14)	47 (2.12)	0.941
Diabetes mellitus	1847 (15.82)	361 (16.26)	0.606
Chronic obstructive pulmonary disease	882 (7.56)	174 (7.84)	0.647
Ischemic heart disease	1431 (12.26)	234 (10.54)	0.022
Stroke	638 (5.47)	160 (7.21)	0.001
Obesity	152 (1.30)	22 (0.99)	0.227
Benign mammary dysplasia	512 (4.39)	28 (1.26)	< 0.001
Other disorders of breast	2078 (17.80)	177 (7.97)	< 0.001
Benign neoplasm of breast	908 (7.78)	76 (3.42)	< 0.001
Mammography			< 0.001
No	6649 (56.97)	1669 (75.18)	
Yes	5023 (43.03)	551 (24.82)	
Time (years)	*N* = 5023	*N* = 551	0.001
≤ 2	3425 (68.19)	338 (61.34)	
> 2	1598 (31.81)	213 (38.66)	
Time (years)	*N* = 5023	*N* = 551	0.005
≤ 2	3425 (68.19)	338 (61.34)	
3–4	355 (7.07)	49 (8.89)	
> 4	1243 (24.75)	164 (29.76)	
Stage			
0	1650 (14.14)		
I	4447 (38.10)		
II	5575 (47.76)		
III		1342 (60.45)	
IV		878 (39.55)	
Index year			0.302
2007–2011	2988 (25.60)	537 (25.81)	
2012–2016	4070 (34.87)	738 (33.24)	
2017–2021	4614 (39.53)	909 (40.95)	

Time: Defined as last mammography to the index date (years). *SD* standard deviation

NT$: New Taiwan Dollar

Low: Less than NT$25,000

Medium: NT$25,000 to NT$39,999

High: NT$40,000 or more

The distribution of chronic kidney disease, diabetes mellitus, chronic obstructive pulmonary disease, and obesity showed no significant difference between early and advanced stages. However, hypertension, hyperlipidemia, chronic liver disease, ischemic heart disease, stroke, benign mammary dysplasia, other breast disorders, and benign neoplasm of the breast were significantly different between the groups. A higher proportion of early-stage patients had undergone mammography (43.03%) compared to advanced-stage patients (24.82%). The proportion of early-stage breast cancer patients with an interval greater than 4 years from the last mammography to the occurrence of breast cancer was 24.75%, while for advanced-stage patients, it was 29.76%, indicating a statistically significant difference between the two groups.

### Delayed mammography and advanced-stage breast cancer

Table 2 presents the relationship between mammography and the odds of advanced-stage breast cancer. Compared with patients who had undergone mammography screening, those who had never undergone screening had higher odds of being diagnosed with advanced-stage breast cancer (aOR = 1.94, 95% CI = 1.73–2.16). The reference group consisted of patients who underwent mammography within the previous two years. Compared with this group, those with an interval of more than two years had higher odds of advanced-stage breast cancer (aOR = 1.42, 95% CI = 1.18–1.70), and those with intervals greater than four years had higher odds (aOR = 1.38, 95% CI = 1.13–1.68). To verify the robustness of the results, sensitivity analyses were conducted. As shown in Supplementary Table 3, after 1:4 matching on age, region, income, and index year, the two groups achieved comparable distributions. Similarly, compared with patients who had undergone mammography screening, those who had never undergone screening had higher odds of being diagnosed with advanced-stage breast cancer. Furthermore, compared with this reference group, patients with screening intervals of more than two years and those with intervals greater than four years also had higher odds of advanced-stage breast cancer (Supplementary Table 4).

**Table 2:** Logistic regression of advanced-stage breast cancer

	cOR (95% CI)	*p*-value	aOR† (95% CI)	*p*-value
Mammography				
Yes	Reference		Reference	
None	2.29 (2.06–2.54)	< 0.001	1.94 (1.73–2.16)	< 0.001
Time (years)				
≤ 2	Reference		Reference	
None	2.54 (2.25–2.88)	< 0.001	2.16 (1.90–2.46)	< 0.001
> 2	1.35 (1.13–1.62)	0.001	1.42 (1.18–1.70)	< 0.001
Time (years)				
≤ 2	Reference		Reference	
None	2.54 (2.25–2.88)	< 0.001	2.16 (1.90–2.45)	< 0.001
3–4	1.40 (1.02–1.92)	0.039	1.57 (1.13–2.17)	0.007
> 4	1.34 (1.10–1.63)	0.004	1.38 (1.13–1.68)	0.002

cOR: Crude odds ratio

aOR: Adjusted odds ratio

†Adjusted for age, hypertension, hyperlipidemia, chronic liver disease, chronic kidney disease, diabetes mellitus, chronic obstructive pulmonary disease, ischemic heart disease, stroke, obesity, benign mammary dysplasia, other disorders of breast, benign neoplasm of breast, and mammography

Time: Defined as last mammography to the index date (years)

**Fig. 2:**
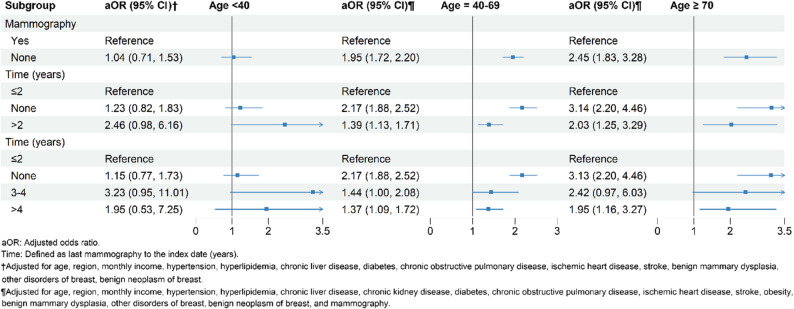
Forest plot of odds ratios for advanced-stage breast cancer among different age period

Figure 2 presents the results of the logistic regression analysis assessing the odds of advanced-stage breast cancer across different age groups. For age under 40, compared with patients who had undergone mammography screening, the absence of mammography was associated with higher odds of advanced-stage breast cancer, with an odds ratio (OR) of 1.04 (95% CI = 0.71–1.53). In the 40–69 age group, compared with patients who had undergone mammography screening, the lack of mammography significantly raised the odds, with an OR of 1.95 (95% CI = 1.72–2.20). Similarly, for aged 70 and older, compared with patients who had undergone mammography screening, the absence of mammography was linked to a higher odd, with an OR of 2.45 (95% CI = 1.83–3.28). Additionally, in the 40–69 age group, compared with patients who had undergone mammography screening within the previous past 2 years, having a mammography with an interval of more than 4 years ago significantly increased the odds (OR = 1.37, 95% CI = 1.09–1.72), as did having a mammography more than 4 years ago in the 70 and older age group (OR = 1.95, 95% CI = 1.16–3.27). A statistically significant interaction was observed between mammography interval and age period (Supplementary Table 5). Stratified analyses showed that the association between screening interval and advanced-stage breast cancer differed across age periods.

**Fig. 3:**
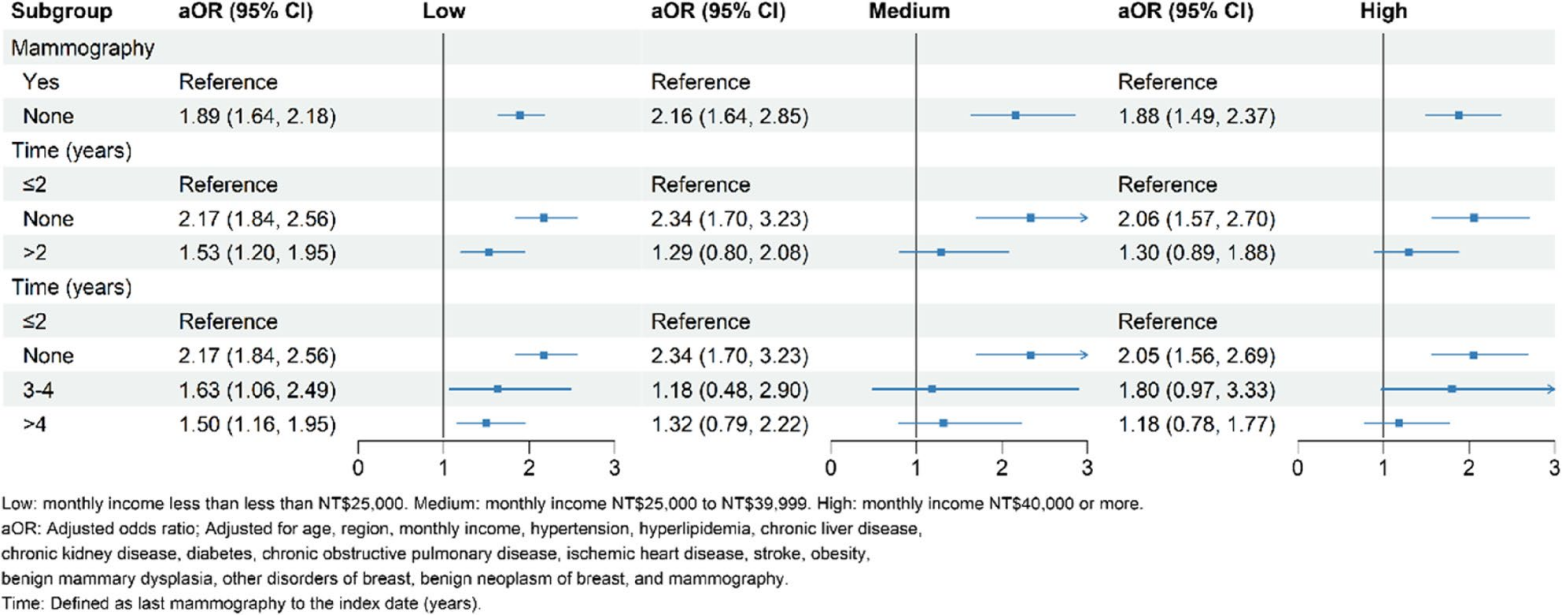
Forest plot of odds ratios for advanced-stage breast cancer among different monthly income

Figure 3 considered the impact of monthly income on the relationship between mammography and advanced-stage breast cancer. In different monthly income groups, compared with patients who had undergone mammography screening, the absence of mammography is associated with a higher odd of advanced-stage breast cancer. Among the low monthly income group, compared with patients who underwent mammography within the previous two years, those whose last mammography was 3–4 years prior had significantly higher odds of advanced-stage breast cancer (OR = 1.63, 95% CI = 1.06–2.49). Similarly, those whose last mammography was more than 4 years prior also had significantly higher odds (OR = 1.50, 95% CI = 1.16–1.95). A statistically significant interaction was not observed between mammography interval and monthly income (Supplementary Table 6).

## Discussion

The study found a significant association between delayed mammography screening and higher odds of being diagnosed with advanced-stage breast cancer. Furthermore, female who did not undergo mammography screening had a substantially higher risk of being diagnosed with advanced-stage breast cancer. In the stratified analysis, female aged 40 and older and those from lower-income groups were associated with higher odds of developing advanced-stage breast cancer due to delayed mammography screening. This study provides the first population-based evidence in Taiwan linking specific mammography screening intervals to advanced-stage breast cancer, with detailed stratification by age and income.

The clinical implications of our findings are notable, as delayed treatment may lead to the development of advanced-stage breast cancer, which complicates treatment and is associated with poorer survival rates [13–15]. Furthermore, patient delays in seeking care for breast abnormalities often exacerbate progression, with longer symptom-to-treatment intervals linked to poorer survival [19]. Although regular screening cannot eliminate all risks, including those from fast-growing tumors such as triple-negative breast cancer (which tends to progress faster), substantial evidence supports mammography’s effectiveness in improving survival among women aged 40–74, with greater benefits in older groups [2, 20]. Consistent adherence to regular screening schedules remains crucial for early detection and reduction of advanced disease and mortality risk [21]. Following American Cancer Society guidelines and maintaining consistent regular screening remain crucial for early detection while significantly reducing the risk of advanced disease, mortality, and late-stage diagnosis due to delayed screening [5]. From a primary care perspective, continuity of care improves adherence to preventive services, including mammography, through timely recommendations, early detection of non-adherence, and mitigation of barriers such as fear or low health literacy [22, 23]. Integrating systematic reminders and prompts into chronic disease management visits offers a practical strategy to enhance screening timeliness in primary care [24].

Systemic healthcare issues, including inadequate resources, long wait times, and access inequalities, also contribute to delayed diagnosis [25]. However, Taiwan’s biennial screening policy and mobile mammography units help minimize regional disparities, as confirmed by our geographic analysis showing no significant differences in stage distribution across regions [26, 27]. Despite the existence of Taiwan’s universal healthcare system and publicly funded biennial screening program for women aged 40–69 years, we still observed significant associations with age and income. This suggests that disparities in screening adherence persist even in a system with broad coverage and organized screening. These findings should be interpreted with caution when generalizing to other countries, particularly those with less comprehensive healthcare coverage or without systematic national screening programs, where socioeconomic disparities may be even larger. In addition, economic hardship remains a major barrier, limiting lower-income individuals’ access to regular check-ups and diagnostics, thereby allowing undetected cancer progression [28–30]. Lower neighborhood-level income was associated with poorer survival outcomes in breast cancer patients, as demonstrated by Ma et al. [31], while lower socioeconomic status was linked to reduced survival rates, according to Taheri et al. and Rutherford et al. [32, 33]. Consistent with these findings, our low-income subgroup exhibited a significantly higher odds of being diagnosed with advanced-stage disease when mammography was delayed beyond four years. Primary care physicians are well-placed to mitigate these barriers by proactively identifying at-risk women and linking them to publicly funded screening programs.

There are several limitations in the current study. First, the use of the National Health Insurance database restricts access to detailed information on personal factors, such as family history of disease, dietary habits, smoking status, alcohol consumption, and physical activity. Consequently, some critical variables influencing breast cancer risk may not be fully accounted for. To address this, we used diagnoses of chronic liver disease and chronic obstructive pulmonary disease as proxies for alcohol consumption and smoking habits, respectively. While these proxies provide partial adjustment for lifestyle-related confounders, they may not fully represent the underlying behaviors and thus could lead to residual confounding. This limitation may affect the internal validity of our findings. Furthermore, due to the involvement of healthcare visits, this database cannot distinguish between mammography used for screening and diagnosis; however, to reduce potential confounding factors, related disease controls such as Benign mammary dysplasia, other breast disorders, and Benign neoplasm of the breast were included. Second, the health insurance database, being claims data, does not provide information on mammography results. This limitation prevents us from adjusting for the time interval between the last mammography and breast cancer diagnosis, regardless of whether the prior screening was normal or abnormal. Additionally, the database restricts access to detailed information on breast cancer subtypes, including Hormone Receptor-Positive (HR+), HER2-Positive, and Triple-Negative (TNBC). This limitation complicates the understanding of genetic factors, such as BRCA mutations, which are essential for personalized treatment and can influence the progression to advanced stages. Third, the categorization of mammography exposure based on the interval since the last screening may not fully capture the nuances of individual screening behavior and adherence. Variations in healthcare access and screening compliance could affect the results. In addition, the study did not account for the potential impact of other breast cancer screening methods, such as ultrasound or MRI, which might be used in conjunction with or as an alternative to mammography. Furthermore, screening-related biases, including lead-time bias (earlier detection without altering disease progression) and length bias (preferential detection of slower-growing tumors), may have contributed to the observed associations. Consequently, the association between delayed mammography and advanced-stage breast cancer may be partially attributable to these factors rather than true differences in disease progression. Fourth, the findings are specific to the Taiwanese population and healthcare system, which may limit their generalizability to other populations with different healthcare systems or socioeconomic conditions.

## Conclusions

This study demonstrates that delayed mammography screening is associated with higher odds of being diagnosed with advanced breast cancer, particularly among women aged 40 and older and those from low-income groups. These findings highlight the need to raise awareness about the benefits of regular mammography and adherence to screening guidelines. Continuity of care can enhance screening adherence through timely recommendations and systematic reminders to promote early detection. Public health efforts should prioritize improving screening participation while strengthening the role of primary care in preventive services to facilitate early detection and better breast cancer outcomes.

## Supplementary Information

Supplementary Material 1.

## Data Availability

The data for this study were obtained from the Health and Welfare Data Science Center and include information from the National Health Insurance Research Database.
