# Supplementary material for: Delayed mammography screening and advanced breast cancer: variation across age and income groups
Source: BMC Public Health. 2026 May 12;26:2054. doi: 10.1186/s12889-026-27731-4 (PMC13335198; doi:10.1186/s12889-026-27731-4)
Supplement: Supplementary file 1 — Supplementary Material 1. [file 12889_2026_27731_MOESM1_ESM.docx]

**Supplementary Table 1** Geographical classification of Taiwan by region

| **Region** | **Areas** |
| --- | --- |
| North | Taipei City, New Taipei City, Taoyuan County, Keelung City, Hsinchu City, Hsinchu County, Miaoli County |
| Central | Taichung City, Changhua County, Nantou County, Yunlin County |
| South | Tainan City, Kaohsiung City, Chiayi City, Chiayi County, Pingtung County |
| East | Yilan County, Hualien County, Taitung County |
| Outlying Islands | Penghu County, Kinmen County, Lienchiang County |

**Supplementary Table 2** Code of comorbidities

| **Item** | **ICD-9-CM** | **ICD-10-CM** |
| --- | --- | --- |
| Hypertension | 401–405 | I10–I15 |
| Hyperlipidemia | 272 | E78 |
| Chronic liver disease | 571 | K70, K73, K74, K75.4, K75.81, K76.0, K76.89, K76.9 |
| Chronic kidney disease | 585 | N184, N185, N186, N189 |
| Diabetes mellitus | 250 | E10, E11, E13 |
| Chronic obstructive pulmonary disease | 491, 492, 496 | J41–J44 |
| Ischemic heart disease | 410-414 | I20–I25 |
| Stroke | 433-438 | I63, I64 |
| Obesity | 278.0 | E66.9 |
| Benign mammary dysplasia | 610 | N60 |
| Other disorders of breast | 611 | N61, N62, N63, N64, N65 |
| Benign neoplasm of breast | 217 | D24 |

**Supplementary Table 3** Demographic characteristics of breast cancer after 1:4 matching by age, region, income, and index year

|  | Early-stage | Advanced-stage | p value |
| --- | --- | --- | --- |
|  | N = 8652 | N = 2163 |  |
| Age |  |  | 0.498 |
| 18-39 | 687 (7.94) | 171 (7.91) |  |
| 40-69 | 6902 (79.77) | 1706 (78.87) |  |
| ≥70 | 1063 (12.29) | 286 (13.22) |  |
| Mean ± SD | 55.66 ± 11.91 | 55.93 ± 12.25 | 0.136 |
| Region |  |  | 0.622 |
| Northern | 4233 (48.93) | 1059 (48.96) |  |
| Center | 1699 (19.64) | 417 (19.28) |  |
| Southern | 2302 (26.61) | 586 (27.09) |  |
| Eastern | 372 (4.30) | 95 (4.39) |  |
| Outlying islands | 46 (0.53) | 6 (0.28) |  |
| Monthly income (NT$) | |  | 0.962 |
| Low | 5979 (69.11) | 1490 (68.89) |  |
| Medium | 1343 (15.52) | 341 (15.77) |  |
| High | 1330 (15.37) | 332 (15.35) |  |
| Comorbidities |  |  |  |
| Hypertension | 2827 (32.67) | 686 (31.72) | 0.394 |
| Hyperlipidemia | 2639 (30.50) | 479 (22.15) | <0.001 |
| Chronic liver disease | 1362 (15.74) | 243 (11.23) | <0.001 |
| Chronic kidney disease | 218 (2.52) | 39 (1.80) | 0.050 |
| Diabetes mellitus | 1560 (18.03) | 332 (15.35) | 0.003 |
| Chronic obstructive pulmonary disease | 697 (8.06) | 159 (7.35) | 0.277 |
| Ischemic heart disease | 1203 (13.90) | 212 (9.80) | <0.001 |
| Stroke | 541 (6.25) | 142 (6.56) | 0.594 |
| Obesity | 117 (1.35) | 20 (0.92) | 0.112 |
| Benign mammary dysplasia | 373 (4.31) | 28 (1.29) | <0.001 |
| Other disorders of breast | 1514 (17.50) | 172 (7.95) | <0.001 |
| Benign neoplasm of breast | 676 (7.81) | 75 (3.47) | <0.001 |
| Mammography |  |  | <0.001 |
| No | 4871 (56.30) | 1629 (75.31) |  |
| Yes | 3781 (43.70) | 534 (24.69) |  |
| Time (years) | N = 3781 | N = 534 | 0.006 |
| ≤2 | 2554 (67.55) | 329 (61.61) |  |
| >2 | 1227 (32.45) | 205 (38.39) |  |
| Time (years) | N = 3781 | N = 534 | 0.022 |
| ≤2 | 2554 (67.55) | 329 (61.61) |  |
| 3-4 | 270 (7.14) | 48 (8.99) |  |
| >4 | 957 (25.31) | 157 (29.40) |  |
| Stage |  |  |  |
| 0 | 1211 (14.00) |  |  |
| I | 3237 (37.41) |  |  |
| II | 4204 (48.59) |  |  |
| III |  | 1309 (60.52) |  |
| IV |  | 854 (39.48) |  |
| Index year |  |  | 0.527 |
| 2007-2011 | 2225 (25.72) | 564 (26.07) |  |
| 2012-2016 | 3015 (34.85) | 726 (33.56) |  |
| 2017-2021 | 3412 (39.44) | 873 (40.36) |  |

Time: Defined as last mammography to the index date (years). SD: standard deviation.

NT$: New Taiwan Dollar.

Low: Less than NT$25,000.

Medium: NT$25,000 to NT$39,999.

High: NT$40,000 or more.

**Supplementary Table 4** Logistic regression of advanced-stage breast cancer after 1:4 matching by age, region, income, and index year

|  | cOR (95% CI) | p-value | aOR† (95% CI) | p-value |
| --- | --- | --- | --- | --- |
| Mammography | |  |  |  |
| Yes | Reference |  | Reference |  |
| None | 2.37 (2.13–2.63) | <0.001 | 1.97 (1.76–2.20) | <0.001 |
| Time (years) | |  |  |  |
| ≤2 | Reference |  | Reference |  |
| None | 2.60 (2.29–2.95) | <0.001 | 2.21 (1.94–2.53) | <0.001 |
| >2 | 1.30 (1.08–1.56) | 0.007 | 1.44 (1.19–1.75) | <0.001 |
| Time (years) | |  |  |  |
| ≤2 | Reference |  | Reference |  |
| None | 2.60 (2.29–2.95) | <0.001 | 2.21 (1.94–2.52) | <0.001 |
| 3-4 | 1.38 (0.99–1.92) | 0.054 | 1.58 (1.13–2.20) | 0.008 |
| >4 | 1.27 (1.04–1.56) | 0.020 | 1.41 (1.14–1.73) | 0.001 |

cOR: Crude odds ratio.

aOR: Adjusted odds ratio.

† Adjusted for age, hypertension, hyperlipidemia, chronic liver disease, chronic kidney disease, diabetes mellitus, chronic obstructive pulmonary disease, ischemic heart disease, stroke, obesity, benign mammary dysplasia, other disorders of breast, benign neoplasm of breast, and mammography.

Time: Defined as last mammography to the index date (years).

**Supplementary Table 5** Odds ratios for advanced-stage breast cancer among different age period

|  | Age <40 |  | Age = 40-69 |  | Age ≥70 |  |  |
| --- | --- | --- | --- | --- | --- | --- | --- |
|  | aOR† (95% CI) | p-value | aOR† (95% CI) | p-value | aOR† (95% CI) | p-value | p for interaction |
| Mammography | |  |  |  |  |  | <0.001 |
| Yes | Reference |  | Reference |  | Reference |  |  |
| None | 1.04 (0.71–1.53) | 0.832 | 1.95 (1.72–2.20) | <0.001 | 2.45 (1.83–3.28) | <0.001 |  |
| Time (years) | |  |  |  |  |  | <0.001 |
| ≤2 | Reference |  | Reference |  | Reference |  |  |
| None | 1.23 (0.82–1.83) | 0.315 | 2.17 (1.88–2.52) | <0.001 | 3.14 (2.20–4.46) | <0.001 |  |
| >2 | 2.46 (0.98–6.16) | 0.055 | 1.39 (1.13–1.71) | 0.002 | 2.03 (1.25–3.29) | 0.004 |  |
| Time (years) | |  |  |  |  |  | 0.002 |
| ≤2 | Reference |  | Reference |  | Reference |  |  |
| None | 1.15 (0.77–1.73) | 0.489 | 2.17 (1.88–2.52) | <0.001 | 3.13 (2.20–4.46) | <0.001 |  |
| 3-4 | 3.23 (0.95–11.01) | 0.061 | 1.44 (1.00–2.08) | 0.049 | 2.42 (0.97–6.03) | 0.058 |  |
| >4 | 1.95 (0.53–7.25) | 0.317 | 1.37 (1.09–1.72) | 0.006 | 1.95 (1.16–3.27) | 0.012 |  |

aOR: Adjusted odds ratio.

Time: Defined as last mammography to the index date (years).

† Adjusted for age, hypertension, hyperlipidemia, chronic liver disease, chronic kidney disease, diabetes mellitus, chronic obstructive pulmonary disease, ischemic heart disease, stroke, obesity, benign mammary dysplasia, other disorders of breast, benign neoplasm of breast, and mammography.

Time: Defined as last mammography to the index date (years).

**Supplementary Table 6** Odds ratios for advanced-stage breast cancer among different monthly income

|  | **Low** |  | **Medium** |  | **High** |  |  |
| --- | --- | --- | --- | --- | --- | --- | --- |
|  | aOR† (95% CI) | p-value | aOR† (95% CI) | p-value | aOR† (95% CI) | p-value | p for interaction |
| Mammography | |  |  |  |  |  | 0.399 |
| Yes | Reference |  | Reference |  | Reference |  |  |
| None | 1.89 (1.64–2.18) | <0.001 | 2.16 (1.64–2.85) | <0.001 | 1.88 (1.49–2.37) | <0.001 |  |
| Time (years) | |  |  |  |  |  | 0.701 |
| ≤2 | Reference |  | Reference |  | Reference |  |  |
| None | 2.17 (1.84–2.56) | <0.001 | 2.34 (1.70–3.23) | <0.001 | 2.06 (1.57–2.70) | <0.001 |  |
| >2 | 1.53 (1.20–1.95) | <0.001 | 1.29 (0.80–2.08) | 0.299 | 1.30 (0.89–1.88) | 0.171 |  |
| Time (years) | |  |  |  |  |  | 0.800 |
| ≤2 | Reference |  | Reference |  | Reference |  |  |
| None | 2.17 (1.84–2.56) | <0.001 | 2.34 (1.70–3.23) | <0.001 | 2.05 (1.56–2.69) | <0.001 |  |
| 3-4 | 1.63 (1.06–2.49) | 0.024 | 1.18 (0.48–2.90) | 0.718 | 1.80 (0.97–3.33) | 0.061 |  |
| >4 | 1.50 (1.16–1.95) | 0.002 | 1.32 (0.79–2.22) | 0.293 | 1.18 (0.78–1.77) | 0.438 |  |

Low: monthly income less than less than NT$25,000. Medium: monthly income NT$25,000 to NT$39,999. High: monthly income NT$40,000 or more.

aOR: Adjusted for age, hypertension, hyperlipidemia, chronic liver disease, chronic kidney disease, diabetes mellitus, chronic obstructive pulmonary disease, ischemic heart disease, stroke, obesity, benign mammary dysplasia, other disorders of breast, benign neoplasm of breast, and mammography.

Time: Defined as last mammography to the index date (years).
